# A Modular Biosensor Design for Quantitative Measurement of Free Nedd8

**DOI:** 10.1021/acssensors.4c01130

**Published:** 2024-09-10

**Authors:** Zachary Wyatt Davis, Korbyn Coyle, Min Kyung Park, Tara Oren, Teagen Hartley, Alyssa Umphlett, Jessilyn Monahan, Kylie Light, Kaylyn Hunter, Yun-Seok Choi

**Affiliations:** School of Natural Sciences, Black Hills State University, Spearfish, South Dakota 57799, United States

**Keywords:** biosensor, Nedd8, FRET, luminescence, nanoBiT, DEN1, neddylation, and deneddylation

## Abstract

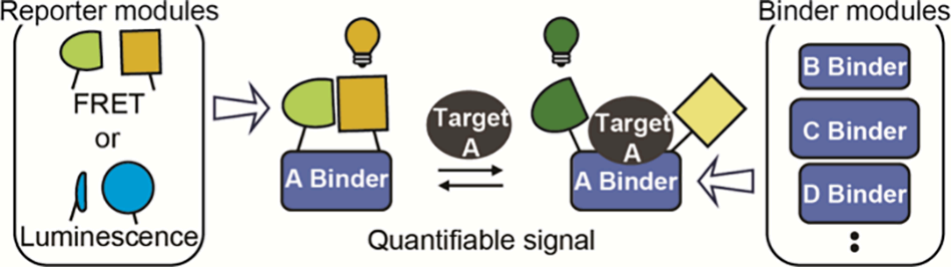

The objective of our study was to develop a genetically encoded biosensor for quantification of Nedd8, a post-translational modifier that regulates cellular signals through conjugation to other proteins. Perturbations in the balance of free (i.e., unconjugated) and conjugated Nedd8 caused by defects in Nedd8 enzymes or cellular stress are implicated in various diseases. Despite the biological and biomedical importance of Nedd8 dynamics, no method exists for direct quantification of free Nedd8, hindering the study of Nedd8 and activities of its associated enzymes. Genetically encoded biosensors are established as tools to study other dynamic systems, but limitations of current biosensor design methods make them poorly suited for free Nedd8 quantification. We have developed a modular method to design genetically encoded biosensors that employs a target binding domain and two reporter domains positioned on opposite sides of the target binding site. Target quantification is based on competition between target binding and the interaction of the reporter domains. We applied our design strategy to free Nedd8 quantification by developing a selective binder for free Nedd8 and combining it with fluorescent or split nanoluciferase reporters. Our sensors produced quantifiable and specific signals for free Nedd8 and enabled real-time monitoring of deneddylation by DEN1 with a physiological substrate. Our sensor design will be useful for high-throughput screening for deneddylation inhibitors, which have potential in treatment of cancers such as acute lymphoblastic leukemia. The modular design strategy can be extended to develop genetically encoded quantitative biosensors for other proteins of interest.

Nedd8 is a ubiquitin-like protein that plays a crucial role in cellular processes through its post-translational conjugation to other proteins. Nedd8 regulates a family of ubiquitin E3 ligases called cullin-RING E3 ligases, which mediate nearly 20% of proteasome-dependent protein degradation in human cells.^1^ Therefore, Nedd8 regulates many pathways.^1,2^

In cells, Nedd8 exists in three “pools”: free (i.e., unconjugated) Nedd8, activated Nedd8, which is linked at its C-terminus through a thioester bond to a neddylating enzyme, and conjugated Nedd8, which is attached through a C-terminal isopeptide bond to a target protein or to another Nedd8 in a poly-Nedd8 chain (Figure 1A).^1^ Nedd8 is conjugated to its targets via a cascade catalyzed by three distinct neddylating enzymes and is deconjugated by a deneddylase (Figure 1A).^1^

**Figure 1:**
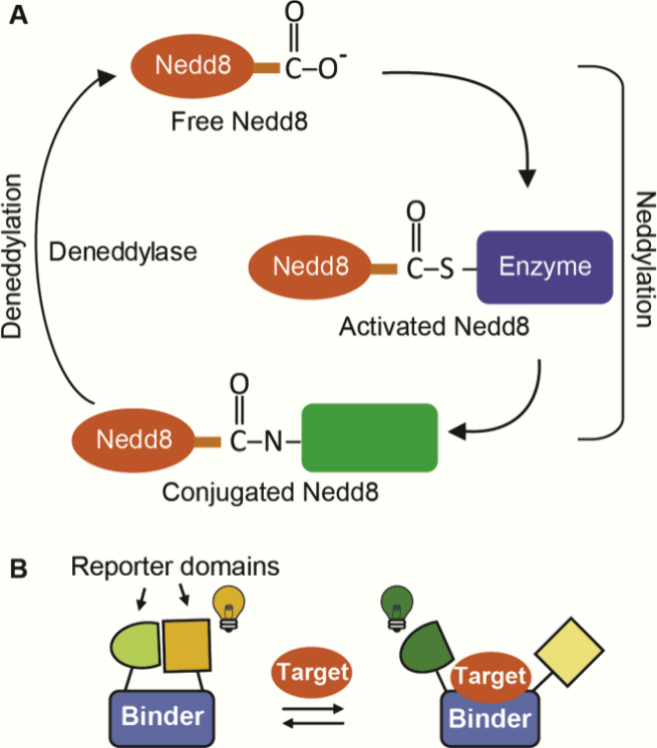
Nedd8 pathway and schematic of the sensor. (A) Neddylation of a substrate protein, a process catalyzed by a cascade of three enzymes (not shown), accelerates ubiquitination activities of the cullin-RING E3 ligases. Deneddylases are responsible for release of free Nedd8 from target proteins. Nedd8 is found in three main forms: (1) free, (2) activated, and (3) conjugated. (B) The sensor consists of a domain that binds specifically to the target (i.e., the binder) and two reporter domains. In the absence of the target, the reporters interact, leading to the generation of a signal. Binding of the target prevents the formation of a functional reporter, resulting in a change in signal intensity, a change in emission wavelength, or both.

The cellular homeostasis of Nedd8 pools is critical for normal function, and defects in neddylation or deneddylation activities can have severe consequences. For instance, neddylating enzymes and deneddylases influence survival and proliferation of some cancer cells, making these enzymes validated targets for cancer treatments.^1,3−6^ The ratio of free Nedd8 to free ubiquitin must also be in balance, because when the concentration of free Nedd8 exceeds that of free ubiquitin or when cells experience extreme stress, ubiquitin ligases incorporate Nedd8 in place of ubiquitin.^7^ This can disrupt ubiquitin-mediated signaling but may also help cells cope with proteotoxic stress.^8^

Despite the importance of Nedd8 pool levels, the tools available for studying Nedd8 dynamics are limited. The only structural feature that distinguishes the Nedd8 forms in the three different pools is the modification at its C-terminus,^1^ and this makes development of methods to selectively monitor individual Nedd8 pools difficult (Figure 1A). Current studies of Nedd8 pools and Nedd8-related enzymes make use of Western blotting or mass spectroscopy; both methods are time and resource intensive, are difficult to scale up, and are poorly suited for dynamics studies or use in vivo.^3,8−10^

For dynamics studies of Nedd8 both in vitro and in vivo, there is a need for new sensors to quantify free Nedd8. Of the Nedd8 pool types, free Nedd8 is an ideal target for measurement for two reasons: First, as a substrate of neddylating enzymes and a product of deneddylases, measurement of free Nedd8 would be far more versatile than a sensor readout of conjugated Nedd8 as it would be generally applicable to studies of Nedd8 pools and to development of Nedd8 enzyme assays. Second, the existence of multiple neddylating enzymes, the enormous diversity of the cullin-RING ligase family, the variety of noncullin substrates, and the presence of poly-Nedd8 chains mean that there are many different kinds of activated or conjugated Nedd8 forms,^1,11^ but there is only one form of free Nedd8.^8^

Genetically encoded biosensors are well-established tools for real-time monitoring of dynamic systems both in vitro and in vivo, and they can be used to provide quantitative data.^12−15^ Unfortunately, current biosensor design strategies are not up to the task of quantifying free Nedd8. Existing designs require a target binding domain (which we refer to here as the binder) that undergoes a major structural change upon target binding to produce a quantifiable signal,^16^ limiting applicability, or require an artificially modified target to enable sensor signaling, potentially compromising physiological relevance.^17^ Furthermore, current biosensor design strategies lack the flexibility to be used at large scales and are not easily generalizable.^18^

To solve this problem, we have developed a modular sensor design strategy that involves a binder that specifically interacts with the target protein and two reporter domains positioned on opposite sides of the target binding site on the binder, thereby allowing a competition between binding to the target and interaction of the reporters (Figure 1B). In the absence of a target, the reporter domains interact; a quantifiable change in this signal results upon interaction with the target. We applied this design method to create two biosensors that selectively bind to free Nedd8. The sensor based on Förster resonance energy transfer (FRET) will be useful in vivo, and the split nanoluciferase sensor, which can monitor deneddylase activity in real time, will enable high-throughput screening of inhibitors of the enzyme for cancer treatment. Importantly, the sensor design method could be used to generate genetically encoded sensors for quantification of other targets.

## Experimental Section

### Cloning

Free Nedd8 in pET3a and precursor Nedd8 in pET28a were gifts from Dr. Robert E. Cohen (Colorado State University at Fort Collins). The mTurquoise2 and mCitrine genes were obtained from Addgene. Gibson assembly (Takara Bio) was used to generate all mutations and to insert reporter domains into eDEN1 to construct eDEN1-FRET and eDEN1-NanoBiT. The cloned gene sequences were confirmed by dideoxy sequencing. The sensor sequences are shown in Figure S1.

### Protein Preparation

All proteins were expressed in BL21-CodonPlus (DE3) *Escherichia coli*. To express and purify free Nedd8 and precursor Nedd8, we used a previously described protocol.^19^ Briefly, the proteins were expressed at 37 °C for 4 h, and cells were lysed. In cell lysates, expressed Nedd8 proteins were found aggregated within inclusion bodies; therefore, protein pellets were dissolved in PBS, pH 7.4, with 8 M urea and were dialyzed against 50 mM Tris, pH 7.5. Nedd8 proteins were purified using Q-Sepharose and SP-Sepharose columns (Cytiva) connected in tandem and then over a Superdex75 size exclusion column. eDEN1 and sensor proteins were expressed at 25 and 7 °C, respectively. Harvested cells were resuspended in PBS, 350 mM NaCl, 10 mM imidazole, and 10 mM β-mercaptoethanol. Cells were lysed by sonication, and insoluble materials were removed by centrifugation at 9999 × *g* at 6 °C for 30 min. Samples were loaded on a HisTrap FF column (Cytiva) equilibrated with the lysis buffer. After washing with lysis buffer, bound proteins were eluted with a gradient of 10 mM to 250 mM imidazole in the same buffer. Fractions containing desired protein were then further purified over an S75 or an S200 size exclusion column (Cytiva) using PBS, pH 7.4, 1 mM dithiothreitol (DTT); for proteins to be labeled with fluorescein, the PBS buffer with 1 mM tris(2-carboxyethyl)phosphine was used instead of 1 mM DTT. Purified proteins were stored at −80 °C. All the column chromatography was performed on an Akta Pure HPLC (Cytiva). Ubiquitin was prepared as described.^20^ Neddylated Cullin1/RBX1 was purchased from Bio-Techne. Protein concentrations were determined based on absorbance at 280 nm, and purity was confirmed by SDS-PAGE.

### Labeling of Nedd8 and Precursor Nedd8

To label free Nedd8 and precursor Nedd8 with fluorescein-maleimide, we mutated threonine at position 20 to cysteine. This Nedd8 was then reacted with fluorescein-maleimide at a 1:3 molar ratio for 2 h at 25 °C. Following this, to quench unreacted fluorescein-maleimide, we added 10 mM β-mercaptoethanol and incubated it for 30 min at 25 °C. A spin column was used to remove unconjugated fluorescein. Degree of labeling (DOL) was calculated using the following equation:
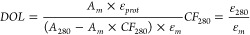

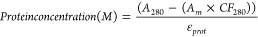
Here, *A*_m_ represents the absorbance at the dye absorption maximum, *A*_280_ is the absorbance of the labeled protein at 280 nm, ε_prot_ is the extinction coefficient of the protein at 280 nm, ε_280_ is the extinction coefficient of the dye alone at 280 nm, ε_m_ is the extinction coefficient at the absorption maximum of the dye, and CF_280_ is the correction factor at 280 nm.

### Binding Assays

Most binding assays were performed in PBS, pH 7.4, 0.05% Brij-35, 0.2 mg/mL ovalbumin, 1 mM DTT; assays with eDEN1-NanoBiT were done without 0.05% Brij-35 and included 70 μM furimazine (AOBIOUS, Cat No: AOB36539). Assays were performed in duplicate or triplicate. To calculate *K*_d_s and signal maxima, the data were fit to a single-site binding model in GraphPad Prism. The fluorescence anisotropy of 4 nM free Nedd8 or precursor Nedd8 labeled with fluorescein was measured in the presence of varying concentrations of eDEN1. The excitation wavelength was 493 nm, and emission was measured at 556 nm using an LS55 fluorescence spectrometer (PerkinElmer). For FRET assays, the fluorescence emission spectra of 50 nM eDEN1-FRET and mutant sensors were measured in the presence of varying concentrations of free Nedd8 or precursor Nedd8. The sample was placed in a 45 μL cuvette (Starna Cells 3-5.45-0-5), and a SpectraMax2 fluorescence spectrometer (Molecular Devices) was employed. The excitation wavelength was 440 nm, and fluorescence spectra were measured from 460 to 560 nm. Luminescence from eDEN1-NanoBiT assays was monitored at 450 nm in a 96-well plate.

## Results and Discussion

### Design Strategy for Genetically Encoded Biosensor for Target Quantification

Our general sensor design employs a binder and two reporter domains that can interact to generate a signal. Target quantification is based on competition between target binding and the interaction of the two reporter domains (Figure 1B): In the absence of the target, the reporters interact, producing a signal. When the target binds to the binder, the reporters are prevented from interacting, resulting in a change in FRET efficiency, fluorescence intensity, or luminescence intensity, depending on choice of reporters. The target can be quantified by comparing the signal to a Nedd8 standard curve. Reporters are attached on opposite sides of the target binding site by flexible peptide linkers. The linkers must be long enough to allow reporters to interact in the absence of the bound target ligand. However, the linker should not be so long that the reporter domains can interact when the target is bound. Our modular biosensor design strategy has important advantages over established biosensor design strategies. In our sensor, it is not necessary that the binder undergo a major structural change upon target binding to elicit a signal; this feature greatly expands options for binders. Furthermore, our method does not require modification of the target, thereby maximizing the physiological relevance of measurements using the sensor.

### Engineered DEN1 Binds Selectively to Free Nedd8

**Figure 2:**
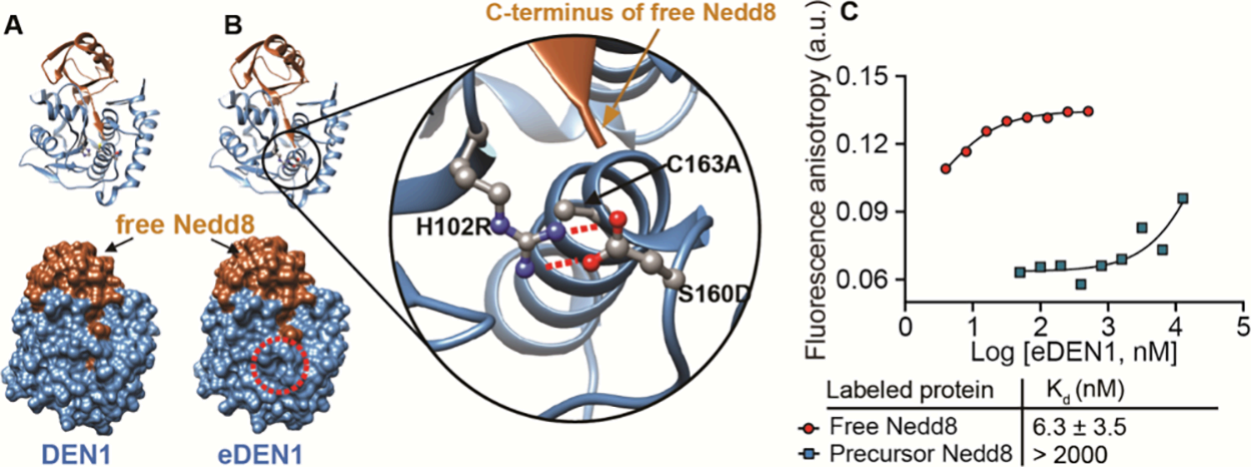
Design and characterization of eDEN1, a free Nedd8 binder. (A) Ribbon (upper) and surface (lower) structures of DEN1 bound to free Nedd8 (PDB ID: 1XT9). (B) Left: ribbon (upper) and surface (lower) structures of eDEN1 bound to free Nedd8. The red dotted circle on the surface structure emphasizes how the mutated amino acids bury the C-terminal residue of free Nedd8 and preclude binding of conjugated substrates. Right: Enlarged view of the mutated amino acids, represented using balls and sticks. Red and blue balls in the side chains represent oxygen and nitrogen, respectively. The red dotted lines represent an ionic interaction. The modeling of mutated amino acids was performed using the Rotamer Library in UCSF Chimera. (C) Fluorescence anisotropy of 4 nM free Nedd8 and precursor Nedd8 labeled with fluorescein measured in the presence of a range of concentrations of eDEN1. All assays were performed twice, and the data points represent average values from duplicate measurements. The fluorescence anisotropy was measured using 493 nm excitation and 556 nm emission. The bottom plateau of the free Nedd8 curve was estimated using the fluorescence anisotropy of free Nedd8 in the absence of eDEN1. The top plateau of the precursor Nedd8 curve was estimated using the top plateau of the free Nedd8 curve. The data were fit to a single-site binding model to calculate *K*_d_.

We used rational design methods to engineer a protein that selectively binds to free Nedd8. The deneddylase DEN1 was selected as a template for the free Nedd8 binder because DEN1 is known to form a complex with Nedd8 and structural information on the DEN1 complex with Nedd8 is available (Figure 2A).^21,22^ As a deneddylase, DEN1 preferentially binds to conjugated Nedd8 over free Nedd8;^23^ therefore, we mutated DEN1 to alter this preference (Figure 2B). First, DEN1 was rendered catalytically inactive by mutating the active site cysteine 163 to alanine (C163A). We then mutated the histidine at position 102 to arginine, and serine at position 160 to aspartic acid; we reasoned that arginine and aspartic acid should form an ionic bond that blocks binding of conjugated Nedd8 (Figure 2B). The resulting construct was named engineered DEN1 (eDEN1) (Figure S1).

To evaluate the specificity of eDEN1 for free Nedd8, we compared its binding to fluorescein-labeled free Nedd8 and, to serve as a mimic of conjugated Nedd8, fluorescein-labeled precursor Nedd8. Precursor Nedd8 is the endogenous form of Nedd8 with an additional five amino acids extending from the C-terminus of free Nedd8 (Figure 1A).^1^ Mutations were introduced at position 20 of both Nedd8 and precursor Nedd8, substituting threonine with cysteine to allow fluorescein labeling. Position 20 of Nedd8 is on the opposite side of the protein from the DEN1 binding site (Figure S2). Binding assays using fluorescence anisotropy with fluorescein-labeled free Nedd8 or fluorescein-labeled precursor Nedd8 showed that eDEN1 has a 6.3 nM *K*_d_ for free Nedd8 and a *K*_d_ greater than 2 μM for precursor Nedd8 (Figure 2C). Thus, eDEN1 binds at least 300-fold better to free Nedd8 than to the precursor form of Nedd8 that has a C-terminal extension. We hypothesize that eDEN1 has a high affinity for free Nedd8 because A163 minimizes the steric hindrance caused by the cysteine’s sulfhydryl group in the active site, allowing the C-terminal carboxylate of Nedd8 to form more hydrogen bonds within the enzyme’s active site, resulting in stronger binding. Morrow et al. reported that an active site cysteine-to-alanine mutation of a deubiquitinase resulted in a high affinity for free ubiquitin through this type of interaction, and that system is similar to the DEN1–Nedd8 interaction.^24^

### FRET Sensor Based on eDEN1 Can Be Used to Quantify Free Nedd8

**Figure 3:**
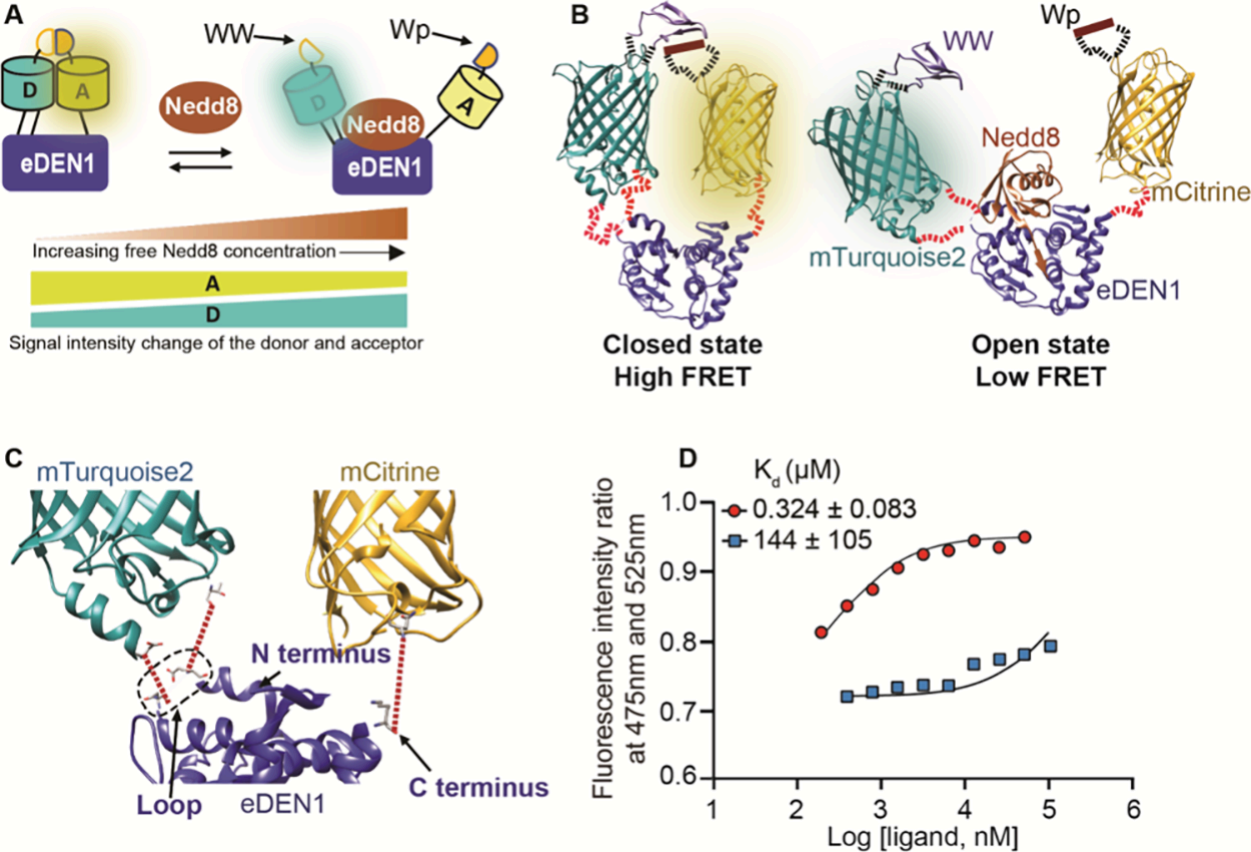
Schematic presentation of free Nedd8 sensing using eDEN1-FRET and characterization of the sensor. (A) Cartoon model of the use of FRET to sense binding of free Nedd8 to eDEN1. D and A represent donor and acceptor proteins, respectively. Binding to free Nedd8 prevents interaction of the reporter domains, causing a simultaneous increase in donor signal intensity and decrease in acceptor signal intensity. (B) Ribbon structures of eDEN1-FRET in the closed state (left) and open state bound to free Nedd8 (right). Domains are connected by flexible linkers shown in red. PDB IDs 1XT9, 3ZTF, 1HUY, and 1JMQ were used for Nedd8-eDEN1, mTurquoise2, mCitrine, and WW structures, respectively. (C) Magnified view of the eDEN1-FRET ribbon structure showing the locations of the loop, N-terminus, and C-terminus. The red dotted lines are measurements of distances used to determine optimal linker lengths. (D) Fluorescence intensity ratio of emission at 475 nm relative to 525 nm of 50 nM eDEN1-FRET-D10N upon titrations with free (red) or precursor (blue) Nedd8. All assays were performed twice, and the data points represent average values from duplicate measurements. The bottom plateau of the free Nedd8 curve was estimated using the ratio data of eDEN1-FRET-D10N in the absence of free Nedd8. The top plateau of the precursor Nedd8 curve was estimated using the top plateau of the free Nedd8 curve. The data were fit to a single-site binding model to calculate *K*_d_.

To turn eDEN1 into a sensor for quantification of free Nedd8, we first used FRET-based reporters, as these are widely used for target quantification in vitro and in vivo.^18^ A key advantage of FRET is that it has two emission peaks, one from the donor fluorescent protein and one from the acceptor, and the ratio of these two signals is independent of sensor concentration (Figure 3A). In our assays, use of the ratio of the two emission peaks gave the sensor a higher dynamic range than use of either signal alone.

We selected mTurquoise2 and mCitrine as donor and acceptor fluorescent proteins, respectively (Figure 3B), because of the high FRET efficiency of this pair.^18^ In our sensor design, the reporters are attached on opposite sides of the Nedd8 binding site to create competition between target binding and the close approach of the two reporters (Figure 3A). Unfortunately, in the three-dimensional eDEN1 structure, the N- and C-termini of eDEN1 are close in space rather than on opposite sides of the Nedd8 binding site, so the fluorescent proteins could not simply be added to each terminus (Figure 3C). To position the reporters on either side of the Nedd8 binding region, the donor fluorescent protein, mTurquoise2, was inserted into a loop positioned opposite the C-terminus, and mCitrine was attached to the C-terminus. To ensure high FRET efficiency, previously characterized intramolecular interacting domains WW and Wp2 were added to loops in the donor and acceptor fluorescent proteins, respectively (Figure 3A).^25,26^ The intramolecular interaction (*K*_d_ = 170 μM) between WW and Wp2 was expected to enhance FRET efficiency when the sensor is not bound to Nedd8 (Figure 3B).

To determine the optimal number of amino acids to be used as linkers between eDEN1 and the donor and acceptor fluorescent proteins, we measured the distances between terminal residues of domains using the UCSF Chimera software (Figure 3C). The number of amino acids obtained from this calculation represents the distance when the linker is fully extended, a state which is unlikely to occur often, so we used a linker with approximately 1.6 times the calculated number of amino acids needed in each linker. Importantly, the linkers were too short to allow the efficient interaction of WW-mTurquoise2 with Wp2-mCitrine when free Nedd8 is bound to eDEN1. The resulting construct was named eDEN1-FRET (Figure S1).

The affinity of eDEN1-FRET for free Nedd8 was determined by monitoring of the emission intensity over the range from 460 to 560 nm upon 440 nm excitation as a function of free Nedd8 concentration. The spectra clearly changed as the free Nedd8 concentration was increased, with an increase of fluorescence intensity at 475 nm and a decrease at 525 nm (Figure S3A). The eDEN1-FRET sensor had a *K*_d_ of 7.61 μM for free Nedd8, which was significantly weaker than that of eDEN1 alone (*K*_d_ = 6.3 nM, Figure 2C).

Although the affinity of eDEN-FRET is in the range appropriate for use in cells,^10^ we sought to further decrease the sensor affinity for free Nedd8. We mutated the aspartic acid at position 10 of the eDEN1 binder to asparagine (D10N); this was shown previously to decrease the affinity of DEN1 for Nedd8.^22^ Surprisingly, this modified sensor, referred to as eDEN1-FRET-D10N, had a higher affinity for free Nedd8 (*K*_d_ = 0.324 μM) than eDEN1-FRET, contrary to our expectations. The spectra of eDEN1-FRET-D10N clearly changed as the free Nedd8 concentration was increased, with an increase of fluorescence intensity at 475 nm and a decrease at 525 nm (Figure 3D). eDEN1-FRET-D10N also showed high selectivity of 444-fold for free Nedd8 compared to precursor Nedd8 (Figure 3D).

eDEN1-FRET-D10N also had an increased dynamic range of signals compared to eDEN1-FRET. We define the signaling dynamic range as ^max^Δ*S*/*S*_0_, where ^max^Δ*S* and *S*_0_ represent the maximum signal change and the signal of a sensor without any target, respectively; the eDEN1-FRET signal was calculated as the ratio between emission peaks at 475 and 525 nm. The dynamic ranges of eDEN1-FRET and eDEN1-FRET-D10N are 0.16 and 0.31, respectively. We speculate that the D10N mutation influences the molecular dynamics of the reporters affecting both affinity of free Nedd8 and the signal dynamic range.

eDEN1-FRET has a micromolar *K*_d_ for Nedd8, making it well-suited to monitor the micromolar range concentrations of the free Nedd8 pool in live cells using its FRET with either radiometric or lifetime readouts. However, the signal dynamic range should be improved for more sensitive quantification of subtle changes in Nedd8 levels under various physiological conditions. The FRET dynamic range could be improved by employing rigid linkers, adjusting the fluorophore orientation using circularly permutated fluorescent proteins, or both.^27,28^ The recently described ChemoG5, which utilizes a HaloTag as its reporter domain, is known to exhibit a high dynamic range in its FRET signal.^29^ We expect that use of ChemoG5 as a reporter would significantly boost the dynamic range of our eDEN1-based biosensor.

To test the effect of the active site mutation C163A on free Nedd8 binding affinity, we created an eDEN1-FRET-D10N construct lacking the C163A mutation. Reversal of the C163A mutation caused a 156-fold decrease in affinity for free Nedd8 (*K*_d_ 50.5 μM) compared to eDEN1-FRET-D10N but increased the dynamic range to 0.42 (Figure S3B). These results confirm that the C163A mutation significantly contributes to the high sensitivity of our binder for free Nedd8. It is possible that different active site substitutions could further tune the sensor’s affinity for free Nedd8. Mutation of eDEN1 may also alter the reporter’s molecular dynamics, resulting in changes in ^max^Δ*S*/*S*_0_.

### The Affinity of Reporter Domain Interaction Affects Dynamic Range and Affinity of eDEN1-FRET for Free Nedd8

As our sensor design is based on competition between the interaction of the reporter domains and the interaction of free Nedd8 with the binder, the affinity of the reporter domain interaction will affect free Nedd8 binding affinity and the ^max^Δ*S*/*S*_0_ of the sensor. To characterize the effect of reporter domain affinity on free Nedd8 binding and dynamic range, we replaced the Wp2 domain on eDEN1-FRET-D10N with Wp1 to increase its affinity for WW. The Wp1 domain has approximately threefold higher affinity for WW than Wp2.^25^ The substitution of Wp2 for Wp1 decreased the ^max^Δ*S*/*S*_0_ to 0.13 from 0.31 (Figure S3C). Substitution of Wp2 for Wp1 also reduced the affinity of the sensor for both free and precursor Nedd8. The affinity for free Nedd8 was decreased by over 18-fold (*K*_d_ = 6.03 μM), and the affinity of precursor Nedd8 for the sensor was significantly decreased (Figure S3C). These data demonstrate that as the strength of the reporter domain interaction increases, free Nedd8 binding is decreased. Therefore, the affinity of the reporters can be used to adjust the affinity of the sensor for the target and the dynamic range of the sensor.

### eDEN1 Can Be Adapted for Use as a Luminescent Reporter

**Figure 4:**
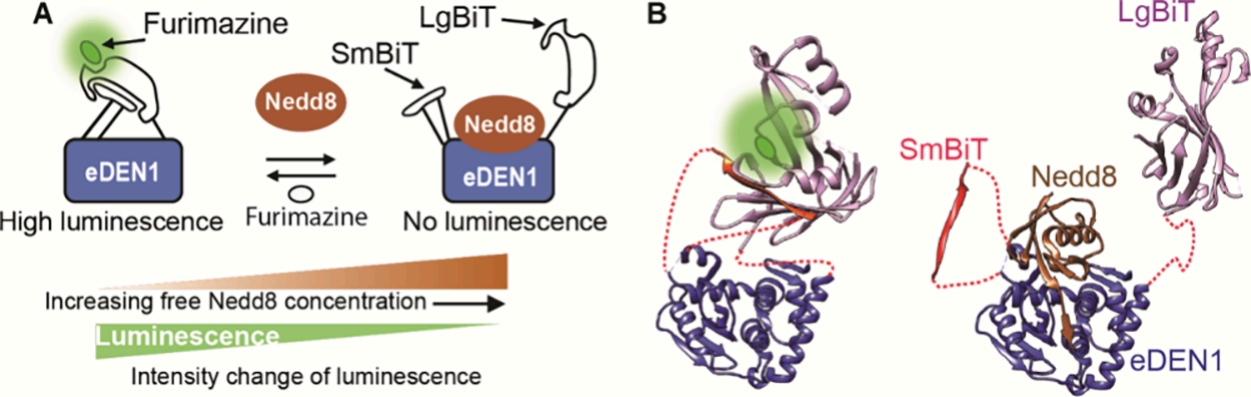
Schematic representation of free Nedd8 sensing using eDEN1-nanoBiT. (A) Cartoon model illustrating the use of eDEN1-nanoBiT to sense binding of free Nedd8. SmBiT and LgBiT represent the small and large subunits of nanoBiT, respectively. Binding to free Nedd8 prevents interaction of the reporter domains, causing a decrease in luminescence intensity. (B) Ribbon structures of eDEN1-nanoBiT in the closed state (left) and open state bound to free Nedd8 (right). The nanoBiT structure is from PDB ID 7SNX. Domains are connected by flexible linkers (red dotted lines).

eDEN1-FRET will be useful for free Nedd8 quantification in cells, but the ^max^Δ*S*/*S*_0_ of the FRET signal may limit its use in assays with purified protein.^28^ A split nanoluciferase (nanoBiT), which is composed of LgBiT and SmBiT fragments, typically has a high signal dynamic range^30^ and is suitable for in vitro assays. We therefore used our modular design strategy to generate a luminescent sensor, eDEN1-nanoBiT. We placed the 11-amino acid SmBiT in the loop of eDEN1, reasoning that it would be less likely to perturb eDEN1 folding than the 17.8 kDa LgBiT. LgBiT was connected to the C-terminus of eDEN1 (Figure 4). Based on our findings with eDEN1-FRET, where a 170 μM *K*_d_ between WW and Wp2 provided the optimal signal dynamic range, we selected versions of SmBiT and LgBiT that showed a *K*_d_ of 190 μM.^30^ SmBiT forms a stretched beta strand when bound to LgBiT (Figure 4B and Figure S4). Therefore, the linkers 1 and 2 connecting SmBiT to the loop of eDEN1 must be long enough to ensure that SmBiT adopts the stretched structure in the bound state with LgBiT (Figure S4A). This eDEN1-nanoBiT sensor was designed to luminesce only in the unbound state.

We cloned and expressed eDEN1-nanoBiT and another version with shorter linkers in *E. coli* (Figures S1 and S4B). We measured luminescence of eDEN1-nanoBiT with and without 150 μM free Nedd8 in the presence of 70 μM furimazine in the *E. coli* cell lysates. eDEN1-nanoBiT showed ^max^Δ*S*/*S*_0_ = 4.7 upon addition of free Nedd8 (Figure S5). The eDEN1-nanoBiT sensor thus has a greater dynamic range than eDEN1-FRET, where ^max^Δ*S*/*S*_0_ = 1.3. In a control experiment, we measured luminescence without added furimazine and detected no signal. The eDEN1-nanoBiT with short linkers showed no luminescence signal with or without free Nedd8 (data not shown); in this construct, SmBiT likely did not adopt the beta-stranded structure, illustrating the importance of the linkers.

### eDEN1-nanoBiT Has a High Specificity and Dynamic Range

**Figure 5:**
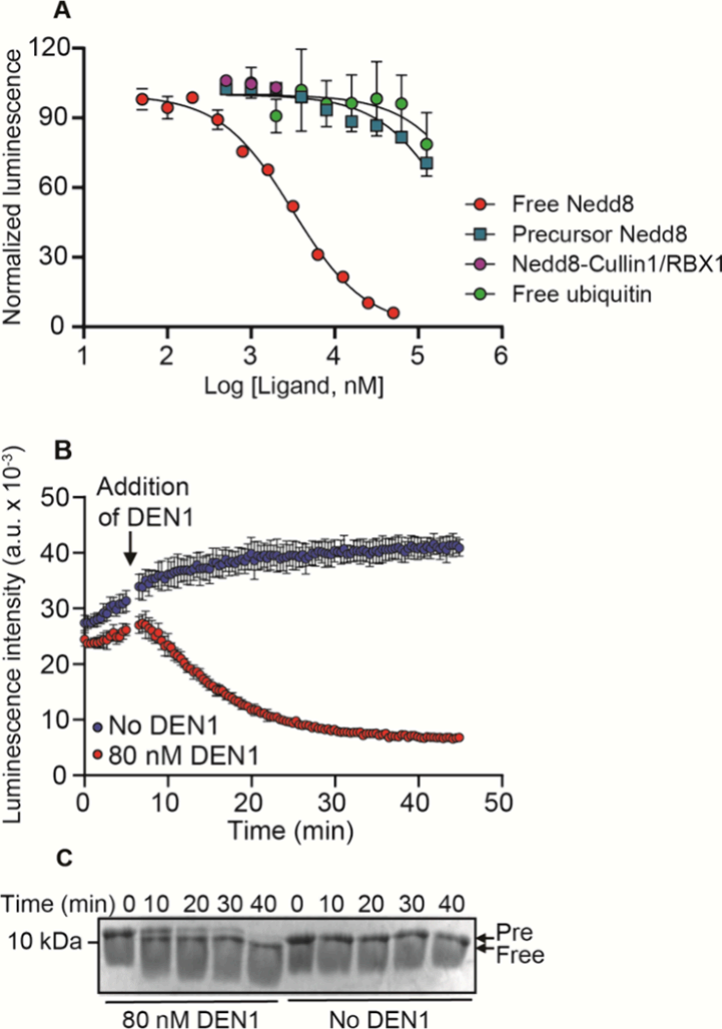
eDEN1-nanoBiT is selective for free Nedd8 and can be used for real-time monitoring of deneddylation. (A) Luminescence (emission at 450 nm) of eDEN1-nanoBiT as a function of the concentration of free Nedd8, conjugated Nedd8, free ubiquitin, and neddylated Cullin1/RBX1 in the presence of 70 μM furimazine at 25 °C. Data from each titration were fit to a single-site binding model using GraphPad Prism software. Binding assays for free Nedd8 and free ubiquitin were performed in triplicate, and others were done in duplicate. The error bars in free Nedd8 and free ubiquitin binding assays show standard deviation. (B) Luminescence of 10 nM eDEN1-nanoBiT at 450 nm was monitored every 10 s in the reaction of 40 μM precursor Nedd8 with and without 80 nM DEN1 at 25 °C. In the reaction with DEN1, 80 nM DEN1 was added 5 min after starting the monitoring. The assay was performed in triplicate. The error bars show standard deviation. (C) Samples analyzed using SDS-PAGE. Pre and free indicate precursor and free Nedd8, respectively.

Analyses of eDEN1-nanoBiT binding to free Nedd8 and precursor Nedd8 showed *K*_d_ values of 3.2 ± 0.7 and 270 ± 94 μM, respectively, establishing selectivity of eDEN1-nanoBiT (84-fold) for free Nedd8 (Figure 5A). The selectivity should be even greater when Nedd8 is conjugated with a bulky substrate, as would be expected with the physiological protein conjugates found in cells. As anticipated, titration of the 2 μM neddylated Cullin1/RBX1 complex, an endogenous conjugated Nedd8,^31^ showed no signal change in the eDEN1-nanoBiT assay (Figure 5A). Although ubiquitin shares a high amino acid sequence identity (60%) with Nedd8, eDEN1-nanoBiT shows high selectivity for Nedd8 relative to ubiquitin (∼200-fold). The ^max^Δ*S*/*S*_0_ of the luminescence signal was ∼10. These data show that eDEN1-nanoBiT has selectivity and a dynamic range that make it useful for monitoring of activities of enzymes that act on Nedd8.

### eDEN1-nanoBiT Monitors Deneddylation in Real-Time Assays

DEN1 is a potential drug target to treat amyotrophic lateral sclerosis (ALS) and chemotherapeutic resistance in acute lymphoblastic leukemia (ALL).^4,32^ Developing an efficient method to monitor DEN1 activity is crucial for discovering DEN1 inhibitors. We therefore tested whether eDEN1-nanoBiT could be used to monitor DEN1 activity. In this assay, 40 μM precursor Nedd8 was incubated with or without 80 nM DEN1 in the presence of 10 nM eDEN1-nanoBiT. As expected, the luminescence intensity of the reaction containing DEN1 decreased over time, indicating that eDEN1-nanoBiT detects the free Nedd8 produced by DEN1-mediated cleavage of precursor Nedd8 (Figure 5B). In contrast, the reaction without DEN1 showed no decrease in luminescence intensity. We further validated the DEN1 activity using SDS-PAGE (Figure 5C). Measuring the luminescence of eDEN1-nanoBiT in a high-throughput drug screen could be used to identify drug candidates that efficiently inhibit DEN1 activity.

This is the first assay developed to monitor deneddylase activities with physiological substrates in real time. Monitoring of deneddylase activities previously has been possible only with artificial substrates such as Nedd8-vinyl sulfone or Nedd8-7-amido-4-methylcoumarin.^33,34^ Use of our biosensor will allow study of deneddylase activities with physiological substrates, thereby providing more physiologically relevant data.

Our sensor design strategy, while potentially generalizable, has limitations. The competitive nature reduces the binder’s affinity for the target. This makes it less suitable for applications requiring extremely tight binding. Additionally, nanoBiT-based sensors have signal instability due to furimazine consumption, potentially decreasing sensitivity and dynamic range if the assay is conducted over several hours. To maintain optimal performance, the assay should be completed within an hour. Although our SDS-PAGE gel data are not necessarily quantitative (Figure 5C), the apparent delayed luminescence response of the sensor to free Nedd8 (observed in comparison of Figure 5B,C) suggests that the sensor may respond slowly to free Nedd8. This might be due to slow on- or off-rates (or both) of free Nedd8 or because the conformational change that brings the reporter domains together is slow. We suspect that the latter explanation is more likely. Nonetheless, this observation indicates that it will be crucial to test whether a selected free Nedd8 sensor can effectively monitor free Nedd8 within the desired time frame when conducting dynamics studies.

## Conclusions

Our modular biosensor design strategy, which does not require major structural changes or artificial modification of the target, allowed the development of genetically encoded biosensors for free Nedd8. The FRET-based free Nedd8 sensor enables real-time monitoring of free Nedd8 dynamics in living cells. The eDEN1-NanoBiT sensor facilitates real-time monitoring of deneddylase DEN1 activity and has the potential to accelerate the identification of DEN1 inhibitors, which could be useful in treating ALS and ALL.

The versatility of our sensor design strategy will allow for easy adaptation to different targets and reporter domains, making it a useful tool for the development of genetically encoded biosensors. As the field of biosensor development continues to evolve, our design strategy has the potential to significantly expand our capacity to create genetically encoded sensors for precise quantification of various targets, ultimately leading to a better understanding of cellular processes and the development of novel therapeutic strategies.

## Supporting Information Available

Primary sequences of the sensors; location of T20 in Nedd8; binding affinities of eDEN1-FRET variants; information on linkers between SmBiT and eDEN1; and evaluation of eDEN1-NanoBiT in *E. coli* cell lysates (PDF)
