# Supplementary material for: A Modular Biosensor Design for Quantitative Measurement of Free Nedd8
Source: ACS Sens. 2024 Sep 10;9(9):4740–7. doi: 10.1021/acssensors.4c01130 (PMC11443517; doi:10.1021/acssensors.4c01130)
Supplement: Supplementary file 1 — se4c01130_si_001.pdf [file se4c01130_si_001.pdf]

Supporting information

for

A modular biosensor design for quantitative measurement of free Nedd8

Zachary Wyatt Davis#, Korbyn Coyle#, Min Kyung Park, Tara Oren, Teagen Hartley, Alyssa Umphlett, Jessilyn Monahan, Kylie Light, Kaylyn Hunter, and Yun-Seok Choi*

School of Natural Sciences, Black Hills State University, South Dakota, 57799, USA

*Corresponding author: yunseok.choi@bhsu.edu

Nedd8

MLIKVKTLTGKEIEIDIEPTDKVERIKERVEEKEGIPPQQQRLIYSGKQMNDEKTAADYKILGGSVLHLVLALRGG

Precursor Nedd8

MPSSHHHHHHSSGLVPRGSHMLIKVKTLTGKEIEIDIEPTDKVERIKERVEEKEGIPPQQQRLIYSGKQMNDEKTAA
DYKILGGSVLHLVLALRGGGGLRQ

eDEN1-FRET

MPSSHHHHHHSSGLVPRGSHMDPVVLSYMDSLLRQSDVSLLDPPSWLNDHIIGFAFEYFANSQFHDCSDHVSFISPE
VTQFIKCTSNPGGSGGMVSKGEELFTGVVPILVELDGDVNGHKFSVSGEGEGDATYGKLTLKFICTTGKLPVPWPTL
VTTLSWGVQCFARYPDHMKQHDFFKSAMPEGYVQERTIFFKDDGNYKTRAEVKFEGDTLVNRIELKGIDFKEDGNIL
GHKLEYNYFSDNVYITADKQKNGIKANFKIRHNIEDGGSTGFEIPDDVPLPAGWEMAKTSSGQRYFKNHIDQTTTWQ
DPRKAGGGGVQLADHYQQNTPIGDGPVLLPDNHYLSTQSKLSKDPNEKRDHMVLLEFVTAAGITLGMGGSGGAEIAM
FLEPLDLPNKRVVFLAINDNSNQAAGGT**H**WSLLVYLQDKNSFFHYDSHSRSNSVHAKQVAEKLEAFLGRKGDKLAFV
EEKAPAQQN**D**YD**A**GMYVICNTEALCQNFFRQQTESLLQLLTPAYITKKRGEWKDLITTLAKTGMVSKGEELFTGVVP
ILVELDGDVNGHKFSVSGEGEGDATYGKLTLKFICTTGKLPVPWPTLVTTFGYGLMCFARYPDHMKQHDFFKSAMPE
GYVQERTIFFKDDGNYKTRAEVKFEGDTLVNRIELKGIDFKEDGNILGHKLEYNYNSHNVYIMADKQKNGIKVNFKI
RHNIEDGSPLPPYTTGGSGSQSGSGTGSESGSTGGSVQLADHYQQNTPIGDGPVLLPDNHYLSYQSKLSKDPNEKRD
HMVLLEFVTAAGITLGMDELYK

eDEN1-FRET_D10N

MPSSHHHHHHSSGLVPRGSHMDPVVLSYM**N**SLLRQSDVSLLDPPSWLNDHIIGFAFEYFANSQFHDCSDHVSFISPE
VTQFIKCTSNPGGSGGMVSKGEELFTGVVPILVELDGDVNGHKFSVSGEGEGDATYGKLTLKFICTTGKLPVPWPTL
VTTLSWGVQCFARYPDHMKQHDFFKSAMPEGYVQERTIFFKDDGNYKTRAEVKFEGDTLVNRIELKGIDFKEDGNIL
GHKLEYNYFSDNVYITADKQKNGIKANFKIRHNIEDGGSTGFEIPDDVPLPAGWEMAKTSSGQRYFKNHIDQTTTWQ
DPRKAGGGGVQLADHYQQNTPIGDGPVLLPDNHYLSTQSKLSKDPNEKRDHMVLLEFVTAAGITLGMGGSGGAEIAM
FLEPLDLPNKRVVFLAINDNSNQAAGGT**H**WSLLVYLQDKNSFFHYDSHSRSNSVHAKQVAEKLEAFLGRKGDKLAFV
EEKAPAQQN**D**YD**A**GMYVICNTEALCQNFFRQQTESLLQLLTPAYITKKRGEWKDLITTLAKTGMVSKGEELFTGVVP
ILVELDGDVNGHKFSVSGEGEGDATYGKLTLKFICTTGKLPVPWPTLVTTFGYGLMCFARYPDHMKQHDFFKSAMPE
GYVQERTIFFKDDGNYKTRAEVKFEGDTLVNRIELKGIDFKEDGNILGHKLEYNYNSHNVYIMADKQKNGIKVNFKI
RHNIEDGSPLPPYTTGGSGSQSGSGTGSESGSTGGSVQLADHYQQNTPIGDGPVLLPDNHYLSYQSKLSKDPNEKRD
HMVLLEFVTAAGITLGMDELYK

eDEN1-FRET_D10N_Wp1

MPSSHHHHHHSSGLVPRGSHMDPVVLSYM**N**SLLRQSDVSLLDPPSWLNDHIIGFAFEYFANSQFHDCSDHVSFISPE
VTQFIKCTSNPGGSGGMVSKGEELFTGVVPILVELDGDVNGHKFSVSGEGEGDATYGKLTLKFICTTGKLPVPWPTL
VTTLSWGVQCFARYPDHMKQHDFFKSAMPEGYVQERTIFFKDDGNYKTRAEVKFEGDTLVNRIELKGIDFKEDGNIL
GHKLEYNYFSDNVYITADKQKNGIKANFKIRHNIEDGGSTGFEIPDDVPLPAGWEMAKTSSGQRYFKNHIDQTTTWQ
DPRKAGGGGVQLADHYQQNTPIGDGPVLLPDNHYLSTQSKLSKDPNEKRDHMVLLEFVTAAGITLGMGGSGGAEIAM
FLEPLDLPNKRVVFLAINDNSNQAAGGT**H**WSLLVYLQDKNSFFHYDSHSRSNSVHAKQVAEKLEAFLGRKGDKLAFV
EEKAPAQQN**D**YD**A**GMYVICNTEALCQNFFRQQTESLLQLLTPAYITKKRGEWKDLITTLAKTGMVSKGEELFTGVVP
ILVELDGDVNGHKFSVSGEGEGDATYGKLTLKFICTTGKLPVPWPTLVTTFGYGLMCFARYPDHMKQHDFFKSAMPE
GYVQERTIFFKDDGNYKTRAEVKFEGDTLVNRIELKGIDFKEDGNILGHKLEYNYNSHNVYIMADKQKNGIKVNFKI
RHNIEDGSGTPPPPYTVGTGGSGSQSGSGTGSESGSTGGSVQLADHYQQNTPIGDGPVLLPDNHYLSYQSKLSKDPN
EKRDHMVLLEFVTAAGITLGMDELYK

eDEN1-FRET_D10N_active site

MPSSHHHHHHSSGLVPRGSHMDPVVLSYM**N**SLLRQSDVSLLDPPSWLNDHIIGFAFEYFANSQFHDCSDHVSFISPE
VTQFIKCTSNPGGSGGMVSKGEELFTGVVPILVELDGDVNGHKFSVSGEGEGDATYGKLTLKFICTTGKLPVPWPTL
VTTLSWGVQCFARYPDHMKQHDFFKSAMPEGYVQERTIFFKDDGNYKTRAEVKFEGDTLVNRIELKGIDFKEDGNIL
GHKLEYNYFSDNVYITADKQKNGIKANFKIRHNIEDGGSTGFEIPDDVPLPAGWEMAKTSSGQRYFKNHIDQTTTWQ
DPRKAGGGGVQLADHYQQNTPIGDGPVLLPDNHYLSTQSKLSKDPNEKRDHMVLLEFVTAAGITLGMGGSGGAEIAM
FLEPLDLPNKRVVFLAINDNSNQAAGGT**H**WSLLVYLQDKNSFFHYDSHSRSNSVHAKQVAEKLEAFLGRKGDKLAFV
EEKAPAQQN**D**YD**C**GMYVICNTEALCQNFFRQQTESLLQLLTPAYITKKRGEWKDLITTLAKTGMVSKGEELFTGVVP
ILVELDGDVNGHKFSVSGEGEGDATYGKLTLKFICTTGKLPVPWPTLVTTFGYGLMCFARYPDHMKQHDFFKSAMPE
GYVQERTIFFKDDGNYKTRAEVKFEGDTLVNRIELKGIDFKEDGNILGHKLEYNYNSHNVYIMADKQKNGIKVNFKI

RHNIEDGSPLPPYTTGGSGSQSGSGTGSESGSTGGSVQLADHYQQNTPIGDGPVLLPDNHYLSYQSKLSKDPNEKRD
HMVLLEFVTAAGITLGMDELYK

eDEN1-NanoBiT with short linker

MPSSHHHHHHSSGLVPRGSHMDPVVLSYM**N**SLLRQSDVSLLDPPSWLNDHIIGFAFEYFANSQFHDCSDHVSFISPE
VTQFIKCTSNPGGSGGSGVTGYRLFEEILGGSGGSGAEIAMFLEPLDLPNKRVVFLAINDNSNQAAGGT**H**WSLLVYL
QDKNSFFHYDSHSRSNSVHAKQVAEKLEAFLGRKGDKLAFVEEKAPAQQN**D**YD**A**GMYVICNTEALCQNFFRQQTESL
LQLLTPAYITKKRGEWKDLITTLAKTGGSGSGGSGMVFTLEDFVGDWEQTAAYNLDQVLEQGGVSSLLQNLAVSVTP
IQRIVRSGENALKIDIHVIIPYEGLSADQMAQIEEVFKVVYPVDDHHFKVILPYGTLVIDGVTPNMLNYFGRPYEGI
AVFDGKKITVTGTLWNGNKIIDERLITPDGSMLFRVTINS

eDEN1-NanoBiT

MPSSHHHHHHSSGLVPRGSHMDPVVLSYM**N**SLLRQSDVSLLDPPSWLNDHIIGFAFEYFANSQFHDCSDHVSFISPE
VTQFIKCTSNPGGSGGSGGGSGGSGSVTGYRLFEEILGSGSGSSGSGSGSGSAEIAMFLEPLDLPNKRVVFLAINDN
SNQAAGGT**H**WSLLVYLQDKNSFFHYDSHSRSNSVHAKQVAEKLEAFLGRKGDKLAFVEEKAPAQQN**D**YD**A**GMYVICN
TEALCQNFFRQQTESLLQLLTPAYITKKRGEWKDLITTLAKTGGSGSGGSGMVFTLEDFVGDWEQTAAYNLDQVLEQ
GGVSSLLQNLAVSVTPIQRIVRSGENALKIDIHVIIPYEGLSADQMAQIEEVFKVVYPVDDHHFKVILPYGTLVIDG
VTPNMLNYFGRPYEGIAVFDGKKITVTGTLWNGNKIIDERLITPDGSMLFRVTINS

Supplementary Figure 1. Primary sequences of Nedd8 and the sensors. The gray, green, brown, pink, yellow, and cyan highlighted regions are eDEN1, mTurquoise2, WW, mCitrine, Wp, and LgBiT, respectively. The boxed sequence is SmBiT. Mutated amino acids are indicated in bold red font. The blue highlighted C in eDEN1-FRET_D10N_active site sequence represents the active cysteine of DEN1.

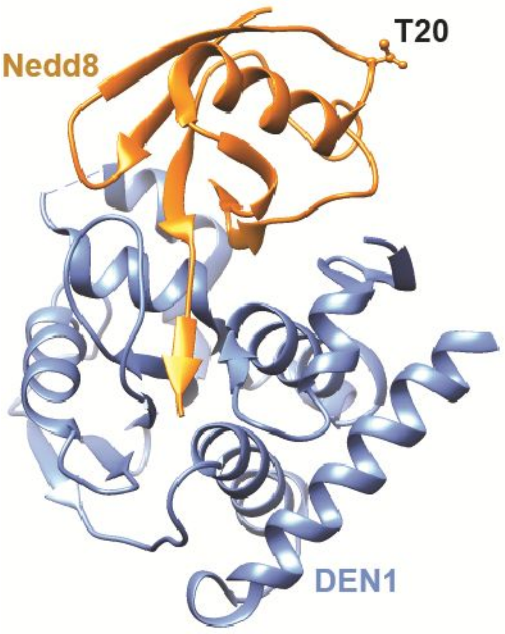

Supplementary Figure 2. Threonine 20 (T20) in Nedd8 is located on the opposite side of the DEN1 binding site. The ribbon structure shows the complex between Nedd8 and DEN1 (PDB ID: 1XT9). For the fluorescein labeling, T20 was mutated to cysteine.

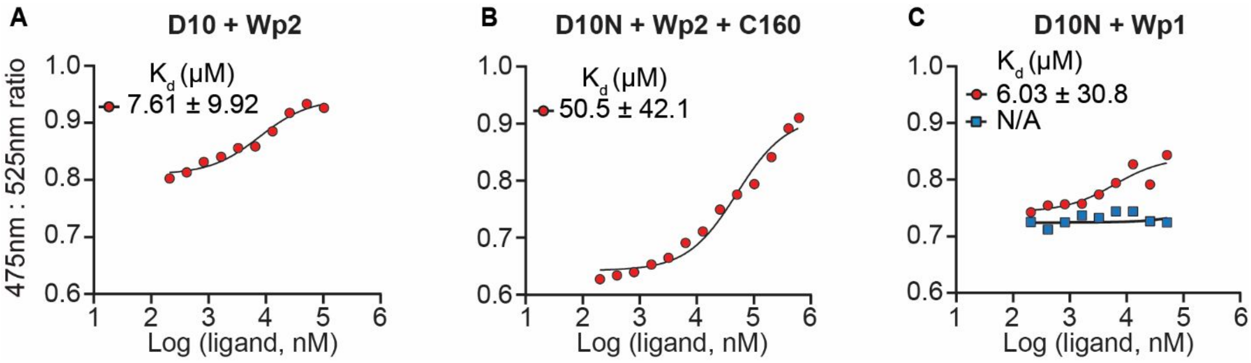

Supplementary Figure 3. Binding affinities of eDEN1-FRET variants. Ratio of emission at 475 nm relative to 525 nm of 50 nM **A)** wild-type eDEN1-FRET, **B)** eDEN1-FRET-D10N lacking the C163A mutation, and **C)** eDEN1-FRET-D10N with Wp2 substituted for Wp1. Sensors were titrated with free (red) or precursor (blue) Nedd8. The data points represent average values from duplicate measurements. Data from each titration were fitted to a single-site binding model using GraphPad Prism software.

A

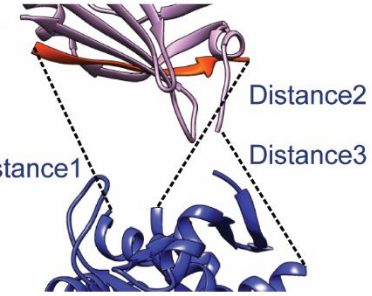

B

Sensor	Distance1	Linker1	Distance2	Linker2	Distance3	Linker3
eDEN1-NanoBiT	32 Å	15aa	32 Å	15aa	22 Å	10aa
eDEN1-NanoBiT with short linkers	32 Å	7aa	32 Å	7aa	22 Å	10aa

Supplementary Figure 4. Information on linkers between SmBiT and eDEN1. A) Magnified view of eDEN1-NanoBiT ribbon structure showing connections between eDEN1 and SmBiT or LgBiT. The black dotted lines are measurements of distances used to determine optimal linker lengths. **B)** Table showing distances and number of amino acids (aa) used for each linker.

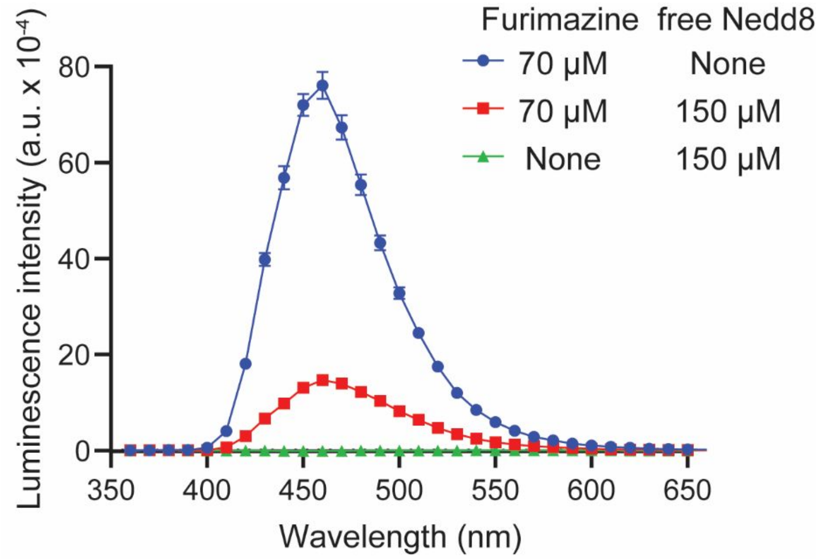

Supplementary Figure 5. Evaluation of eDEN1-NanoBiT in *E. coli* cell lysates. *E. coli* cells expressing eDEN1-NanoBiT were lysed, and the cell lysate was mixed with 70 μM furimazine and 150 μM free Nedd8. Luminescence intensity was measured from 350 to 650 nm in a 96-well plate using a SpectraMax2 fluorescence spectrometer. The assays were performed in duplicate.
